# Cryptic genetic variation in an inbreeding and cosmopolitan pest, *Xylosandrus crassiusculus*, revealed using ddRADseq

**DOI:** 10.1002/ece3.3625

**Published:** 2017-11-12

**Authors:** Caroline Storer, Adam Payton, Stuart McDaniel, Bjarte Jordal, Jiri Hulcr

**Affiliations:** ^1^ School of Forest Resources and Conservation Institute of Food and Agricultural Sciences University of Florida Gainesville FL USA; ^2^ Biology Department University of Florida Gainesville FL USA; ^3^ University Museum University of Bergen Bergen Norway; ^4^ Department of Entomology and Nematology Institute of Food and Agricultural Sciences University of Florida Gainesville FL USA

**Keywords:** ambrosia beetle, ddRADseq, inbreeding, invasive species, population structure, single‐nucleotide polymorphisms

## Abstract

Each year new exotic species are transported across the world through global commerce, causing considerable economic and ecological damage. An important component of managing invasion pathways is to identify source populations. Some of the most widespread exotic species are haplodiploid ambrosia beetles. The ability to mate with siblings (inbreed) and their transportable food source (symbiotic fungus) have enabled them to colonize most of the world and become pests of plant nurseries, lumber, and forests. One of the fastest spreading ambrosia beetles is *Xylosandrus crassiusculus*. In order to discover the source populations of this globally invasive species, track its movement around the world, and test biogeographical scenarios, we combined restriction site‐associated DNA sequencing (RADseq) with comprehensive sampling across the species native and introduced range. From 1,365 genotyped SNP loci across 198 individuals, we determined that in its native range, *X. crassiusculus* is comprised of a population in Southeast Asia that includes mainland China, Thailand, and Taiwan, and a second island population in Japan. North America and Central America were colonized from the island populations, while Africa and Oceania were colonized from the mainland Asia, and Hawaii was colonized by both populations. Populations of *X. crassiusculus* in North America were genetically diverse and highly structured, suggesting (1) numerous, repeated introductions; (2) introduction of a large founding population; or (3) both scenarios with higher than expected outcrossing. *X. crassiusculus*, other wood‐boring insects, and indeed many other pests with unusual genetic structure continue to spread around the world. We show that contemporary genetic methods offer a powerful tool for understanding and preventing pathways of future biosecurity threats.

## 1 INTRODUCTION

Inference from genetic population structure is fundamental to many biological disciplines. In an era of global commerce, it is also increasingly critical for determining the sources and pathways of organisms that cause harm to humans and ecosystems (Hulme, 2009). Such information is essential for international regulation of commerce and for assessing future biosecurity threats.

Our ability to infer the history of populations used to be restricted by the availability of molecular markers, which were historically difficult and expensive to develop (Avise, 2012). However, with the advent of high‐throughput sequencing and genotype‐by‐sequencing strategies such as restriction site‐associated DNA sequencing (RADseq), marker discovery and genotyping have proliferated. The ease and accessibility of RADseq have facilitated studies of ecological and evolutionary genetics in nonmodel organisms (Andrews, Good, Miller, Luikart, & Hohenlohe, 2016; Narum, Buerkle, Davey, Miller, & Hohenlohe, 2013) including exotic species with unknown population histories (Garnas et al., 2016; Hasselmann, Ferretti, & Zayed, 2015).

The Xyleborini ambrosia beetles (Coleoptera: Curculionidae: Scolytinae) are some of the most widespread invasive insects (Haack, 2011). Their success has been facilitated by two features: the farming of a fungal symbiont as a food source and their haplodiploid reproductive strategy, characterized by high inbreeding (Jordal, Beaver, & Kirkendall, 2001). Increasingly, these exotic ambrosia beetles are becoming pests of plant nurseries, stored lumber, and forests (Hulcr & Dunn, 2011). Despite their importance, the population structure of globally distributed Xyleborini beetles has rarely been studied (Gohli, Selvarajah, Kirkendall, & Jordal, 2016), and the effect of their unique biology on population genetic structure remains unknown (Kirkendall, Biedermann, & Jordal, 2015).

Many extraordinarily invasive insects possess extraordinary genetic and biological features. For example, haplodiploidy and/or female‐biased rapid reproduction is disproportionately represented among globally invasive pests (Ehrlich, 1986). Examples include sap sucking Heteroptera with female‐biased reproduction and nonassortative mating (Liu et al., 2007) or ants with haplodiploidy and inbreeding which allows incipient populations to prosper even in the absence of genetic variability (Holway, Suarez, & Case, 1998).

An example of an invasive group that combines several unusual genetic features is the Xyleborini ambrosia beetles, a group of haplodiploid, highly inbred fungus farming wood borers. Perhaps the most widely spread Xyleborini species is the Asian granulated ambrosia beetle *Xylosandrus crassiusculus* (Flechtmann & Atkinson, 2016). This species is assumed to have originated in Asia and has been introduced throughout the tropics and subtropics likely via human‐mediated means. Within the last several decades, it has reached all tropical and subtropical areas of the world, and it often becomes the dominant ambrosia beetle species in each newly colonized area. In the United States, for example, it was first recorded only 42 years ago and quickly became one of the most commonly collected ambrosia beetles in eastern North America (Miller & Rabaglia, 2009). Not only does it dominate natural communities, but it is also becoming a serious pest in the nursery industry. The beetle is highly attracted to volatiles from stressed and weakened trees and is able to precisely locate such trees in a nursery (Ranger, Reding, Persad, & Herms, 2010). Minor stresses, such as temporary flooding or late frost, used to be of little concern in nurseries because these trees typically recovered. Now, with *X. crassiusculus* ubiquitous in the landscape, stressed trees are rapidly colonized and killed (Ranger, Schultz, Frank, Chong, & Reding, 2015), and nurseries are forced to adapt their management or assume significant losses.

Despite the impressive speed of the *X. crassiusculus* global invasion, nothing is known about its population structure and spread. The number of introductions and their origins, the level of inbreeding, and any possible cryptic diversity remain unknown. The only available data on the population genetics of *X. crassiusculus* are from Dole et al. (Dole, Jordal, & Cognato, 2010), who used cytochrome oxidase I mtDNA sequencing (the DNA “barcode”) to infer that the population in the United States is clonal, while genetic distances between other populations are large and analogous to distances observed in interspecific comparisons.

In order to discover the source populations of the invasive *X. crassiusculus* and track its human‐assisted movement around the world, we used RADseq. The method combines high‐throughput genomic marker discovery and genotyping, which is valuable in a system with few molecular markers. The RADseq approach is combined here with a comprehensive sample of the world's populations of *X. crassiusculus*, which enabled us to test, for the first time, specific hypotheses on the global spread of an inbreeding pest: Is there a single source population or multiple sources? Is the intraspecific genetic divergence of such rapidly spreading species low in invaded regions (suggesting a series of single introductions) or high (suggesting the global movement of multiple populations)? And, given that this is a highly inbred and haplodiploid species, will RADseq uncover population genetic structure expected from studies of regularly reproducing invasive species?

## 2 METHODS

### 2.1 Specimens and sample preparation

**Figure 1:**
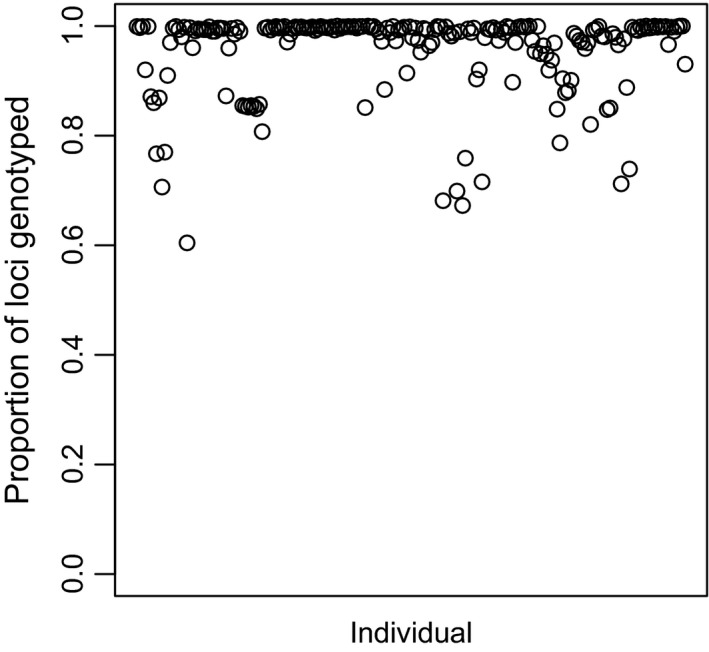
Proportion of genotyped single‐nucleotide polymorphisms per individual

We sampled 198 female *X. crassiusculus* beetles from the species' native and introduced range for genotyping‐by‐sequencing (Table 1, Figure 1). All specimens had been stored at −80°C in 95% EtOH in the University of Florida Forest Entomology Collection at the School of Forest Resources and Conservation. Only females were used because males are rare in species with the haplodiploid system. In order to minimize nontarget species DNA carryover, all specimens were surface washed by vortexing in a PBS‐Tween solution. Additionally, each beetle was dissected to remove the digestive tract, mycangium (the fungus storing organ), and the spermatheca (a female organ that stores sperm). DNA was then extracted from the remaining beetle muscle tissue using a Qiagen DNeasy Blood and Tissue Kit per the manufacturer's instructions. DNA's quantity and quality were assessed using semiquantitative gel electrophoresis. After dissection and DNA extraction, no voucher material remained.

**Table 1:** Sample location and sample sizes with the origin of populations at each location, the first reference for establishing origin, and pest status

Continent	Country	Locality	*n*	Origin	Status	Reference and notes
Africa	Cameroon	Limbe	7	Introduced	Unknown	Hagedorn 1908 (syn. *X. mascarenus*); first detection in Africa
	Ghana	Ankasa	11	Introduced	Unknown	Hagedorn 1908 (syn. *X. mascarenus*); first detection in Africa
	Madagascar	Ranomafana	8	Introduced	Unknown	Hagedorn 1908 (syn. *X. mascarenus*); first detection in Africa
Asia	China	Xishuangbanna	20	Native	Nonpest	Yin et al. 1984
	Indonesia	East Java	11	Native	Unknown	Blandford 1896 (syn. *X. semigranosus*)
	Japan	Aichi	6	Native	Nonpest	Eichhoff 1878 (syn. *X. semiopacus*)
		Iriomote	1	Native	Nonpest	Eichhoff 1878 (syn. *X. semiopacus*)
		Okinawa	12	Native	Nonpest	Eichhoff 1878(syn. *X. semiopacus)*
	Taiwan	Dali	6	Native	Nonpest	Murayama 1934
	Thailand	Chiang Mai	4	Native	Nonpest	Beaver & Browne 1975
Central America	Honduras	Atlántida	3	Introduced	Unknown	CG Storer 2013 (unpublished) ; first detection
North America	United States	Arkansas	5	Introduced	Pest	EG Riley 1996 (unpublished)
		Florida	24	Introduced	Pest	Deyrup & Atkinson 1987
		Georgia	22	Introduced	Pest	TH Atkinson 2008 (unpublished)
		Hawaii	7	Introduced	Pest	Samuelson 1981
		Maryland	8	Introduced	Pest	Rabaglia 2003
		North Carolina	12	Introduced	Pest	Chapin & Oliver 1986
		South Carolina	4	Introduced	Pest	Anderson 1974; first detection in United States
		Virginia	20	Introduced	Pest	Rabaglia et al.^2006^
Oceania	Papua New Guinea	Mandang	7	Introduced^a^	Unknown	Wood & Bright 1992

Origin of each beetle population at each location is as reported by the Centre of Agriculture and Biosciences (CABI) unless otherwise noted. Pest status designation is as reported by the European and Mediterranean Plant Protection Organization (EPPO).

a See (Hulcr & Cognato, 2013) for discussion of a recent introduction into Papua New Guinea.

### 2.2 ddRAD sequencing

Specimens with at least 160 ng of nondegraded DNA were prepared for double‐digest restriction site‐associated sequencing (ddRADseq Peterson, Weber, Kay, Fisher, & Hoekstra, 2012). Following (Parchman et al., 2012), DNA was digested with the rare EcoRI and common MseI restriction enzymes resulting in fragments with sticky ends. Illumina sequencing adaptors and custom barcodes (Parchman et al., 2012) were ligated to the EcoRI terminus sticky end with a common adaptor ligated to the MseI terminus of the digestion product, and then the adaptor‐ligated fragments were PCR amplified using primers for Illumina sequencing adaptors. Each adaptor+barcode was composed of an Illumina adaptor, a unique barcode between 8 and 14 bases in length that varied by at least four bases, and the restriction site. Amplified digestion‐ligation products were visually inspected using gel electrophoresis and then pooled in equal volumes for sequencing. Gel‐based size selection for 200–500 bp fragments and sequencing library purification using Agencourt AMPure XP beads (Beckman Coulter) were performed at the UF ICBR core‐sequencing laboratory (http://www.biotech.ufl.edu/). The size‐selected and purified sequencing library was further pooled with a similarly constructed library consisting of 96 bar‐coded samples and then loaded on the Illumina NextSeq500 high‐output flow cell for a shared 150‐cycle sequencing run. Unfiltered demultiplexed sequences were deposited in NCBI GenBank Short Read Archive (Accessions: SRR4181068‐265).

### 2.3 Read processing and genotyping

Illumina adaptor sequences were automatically trimmed from all reads by Illumina's BaseSpace software. All subsequent steps were conducted in the UF High‐Performance Computing instance of GALAXY (Blankenberg et al., 2001; Giardine et al., 2005; Goecks, Nekrutenko, & Taylor, 2010). Trimmed reads were quality‐filtered using the FASTX (http://hannonlab.cshl.edu/fastx_toolkit/) toolkit for a Phred score of 20 across 90% of the read. Quality‐filtered reads were demultiplexed using the FASTX Toolkit “barcode splitter.” Barcodes and restriction site nucleotides were then trimmed from the beginning of the read using “FASTX trimmer” from the FASTX Toolkit. Due to variation in read length, all reads were trimmed to 71 bases. This maximized the number of reads recovered per individual because downstream processing required that all reads be the same length per individual. The resulting FASTA files for each individual were processed using STACKS v. 1.24 (Catchen, Hohenlohe, Bassham, Amores, & Cresko, 2013) for locus discovery and genotyping. Using STACKS *denovo_map.pl* script, RAD tags were first assembled into de novo loci per individual in “ustacks”; from these loci, a catalog of all possible loci was assembled in “cstacks”; and then, individuals were compared to the catalog in “sstacks”. Optimum parameters for loci and catalog assembly were determined following recommendations from (Mastretta‐Yanes et al., 2014). Stack and loci assembly parameters were as follows: a minimum of three reads for stack creation (‐m 3), three allowed mismatches between stacks during loci assembly (‐M 3), and an allowance of two mismatches between loci when building the catalog (‐n 2). While three mismatches within an individual allowed for up to 4.2% genetic divergence, they were chosen to maximize the number of shared loci detected, increase the probability of detecting presumably rare heterozygous loci, and reduce the chances of creating erroneous loci.

Single‐nucleotide polymorphism (SNP) genotypes were generated from the output of *denovo_map.pl* using the “populations” module of STACKS. In STACKS “populations,” a locus was retained if it was shared by 90% of the 198 individuals sequenced (‐r 0.9), there was a minimum of nine reads per individual at a locus (‐m 9), and a minimum allele frequency of 0.05 per locus (‐a 0.05). The additional parameter that only a single SNP should be called per a read was specified using –write_single_snp. We reduced the potential confounding effect of the fungal sequence carryover in two ways: by removing the mycangium as described above and bioinformatically. Catalog loci were compared to a draft genome of *Ambrosiella roeperi* (the specific mutualist of *X. crassiusculus*; D Vanderpool and JP McCutcheon, unpublished). Loci that aligned to the fungus genome with 99% similarity across the entire read were excluded from SNP calling by specifying them as blacklisted loci (‐B) in STACKS “populations.”

### 2.4 Population structure and history

**Figure 2:**
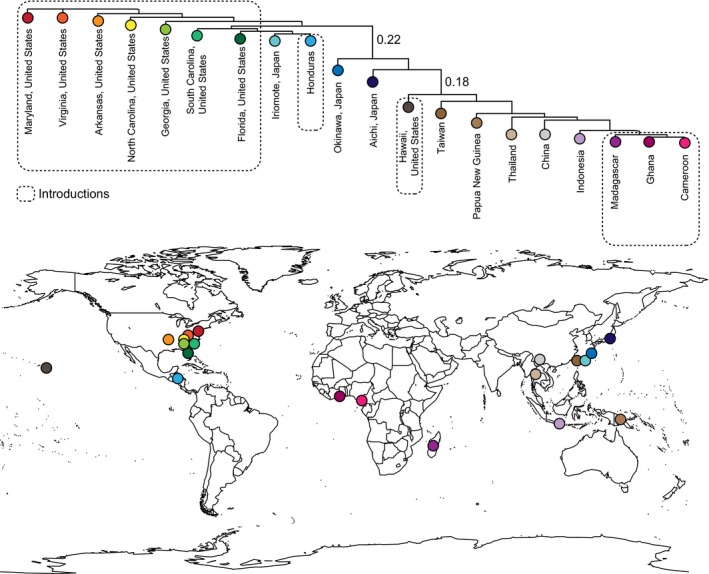
Sampling locations with a neighbor‐joining tree of average genetic distance between locations. Genetic distances >0.1 are shown

Data transformation and treatment of missing genotypes were performed in R version 3.2.1 (R Core Team 2015) using the function tab from the package *adegenet* (Jombart, 2008). Genotypes were standardized and transformed into relative allele frequencies. Missing alleles (4.46%; Figure 2) were replaced by the mean locus‐specific allele frequency. This treatment of missing data is recommended for downstream multivariate analyses. Any biases introduced are minimal given a low number of missing genotypes and lack of systematic missing data at specific loci or for specific individuals. Basic population and summary statistics were generated from STACKS “populations” sumstats.summary output, using the *adegenet* summary function and the *hierfstat* basic.stats function. Observed heterozygosity and inbreeding index *F*_IS_ were used to assess the extent of inbreeding as indicated by reduced heterozygosity. Additionally, the proportion of shared alleles between individuals was calculated for the detection of clones. A Euclidian distance matrix was generated with *stats* dist for downstream multivariate analyses. Absolute genetic distance between locations was calculated in *adegenet* using dist.genpop and was visualized as a neighbor‐joining tree using *ape* nj.

Hierarchical clustering was used to associate nonnative beetles with native beetle populations and to determine the relationships between and among groups. This clustering method was deemed more suitable than nonhierarchical clustering because population substructure is likely when sampling across a global metapopulation. All hierarchical clustering was performed using *stats* hclust. The suitability of different fusion methods (single, complete, centroid, median, UPGMA, and Ward's) was assessed using the cophenetic correlation coefficient. First, the cophenetic distances for hierarchical clustering using each fusion method were calculated using the function cophenetic {*stats*}. Then, the cophenetic distances were correlated with the original distance matrix with *stats* cor. Dendrograms built using all five methods preserved the original distance matrix similarly well (*c* > 0.92) with complete linkage and average linkage (UPGMA) performing the best (*c* = 0.98). Similar hierarchical relationships were observed using complete linkage and average linkage; therefore, the space‐dilating complete linkage method was used for downstream analyses. Multiscale bootstrap resampling with 10,000 replicates was performed with *pvclust* package function pvclust to calculate *p*‐values for hierarchical clusters.

While hierarchal clustering was deemed most suitable for the inference of global population connections, Bayesian clustering in STRUCTURE was used to infer shared ancestry and probable cluster numbers (Pritchard, Stephens, & Donnelly, 2000). STRUCTURE was run with a burn‐in of 1,00,000 generations with 1,50,000 subsequent generations in quintuplicate for *K* = 1 through *K* = 16, allowing for admixture. Change in log‐likelihood scores between *K* (Evanno, Regnaut, & Goudet, 2005) was used to determine the most probable number of clusters with STRUCTURE HARVESTER (Earl & vonHoldt, 2012).

Genetic differentiation between clusters (determined here) and locations was tested by hierarchical analysis of molecular variance (AMOVA) in poppr.amova {*poppr*}. The extent of genetic differentiation was also estimated using phi‐statistics within poppr.amova. The significance of variance components, such as Φ_CT_, differentiation between clusters; Φ_SC,_ differentiation among locations within clusters; and Φ_ST_, differentiation among locations, was assessed using a permutation test implemented through randtest {*ape4*} with 9,999 permutations. Fixation indices between locations, pairwise F_ST_, were calculated in *adegenet* using the *hierfstat* wrapper function pairwise.fst.

Group membership probabilities for the clusters identified in hierarchical cluster analysis and for geographic location were determined using a Discriminant Analysis of Principal Components (DAPC), which has no model assumptions (Jombart, Devillard, & Balloux, 2010). To determine the number of principal components to retain for the analysis, the *a*‐score, which is the difference between observed discrimination and random discrimination, was calculated using *adegenet* a.score. Additional assessment of the optimum number of PCs to retain for the DAPC was performed through cross‐validation by subsetting the data into training and validation sets using xvalDapc. After the number of principal components was determined, the DAPC was performed using for a specified number of clusters (hierarchical and location) using the function dapc, and the probability of individual membership to each cluster was visualized using compoplot.

## 3 RESULTS

### 3.1 Sequencing and genotyping

Using ddRADseq, we successfully sequenced over 11 billion bases of DNA for SNP discovery and genotyping in *X. crassiusculus*. An average of 6,63,917 reads (±1,70,404 *SD*) was sequenced per individual, and 2,29,976 putative loci were identified. Mapping these putative loci to the fungal symbiont genome *Ambrosiella roeperi* resulted in the removal of 16% of loci suggesting that dissection and in silico removal of fungal symbiont reads were successful. Only 4,807 (2.5%) of the remaining putative loci were shared among 90% of the 198 beetles sequenced and had a minimum of nine reads per individual. Among the shared loci, 28.4% contained single‐nucleotide polymorphisms (SNPs or variant sites). Although 3,844 SNPs were identified, many of these SNPs were in the same locus (read), therefore only one SNP per locus was considered for genotyping which resulted in a total of 1,365 SNPs. Of the genome sampled here using ddRADseq 1.1% contained variation when all SNPs (variant sites) across all shared loci are considered, and 0.3% when only genotyped SNPs are considered.

Average homozygosity (0.99 ± 0.02) and inbreeding coefficient *F*_IS_ (0.84) were high for the 1,365 genotyped SNPs (variant sites) and at most geographic locations (Table 2) using all three population summary statistics programs: STACKS “populations,” *adegenet*, and *hierfstat*. Estimates of these indices may be impacted by locus assembly parameters, such as overmerging of loci during assembly. However, heterozygote genotypes remained rare (Table 3). Additionally, more stringent mismatch parameters were tested for locus discovery/assembly and resulted in significant reduction in shared loci with no change in population summary statistics.

**Table 2:** Summary statistics for RAD loci at each locality

Country	Locality	*n*	Single‐nucleotide polymorphisms	Gene diversity	Shared alleles	Observed heterozygosity	*F*_IS_
Cameroon	Limbe	7	3	0.0006	1.00	0.0005	0.28
Ghana	Ankasa	11	3	0.0005	1.00	0.0004	0.41
Madagascar	Ranomafana	8	1	0.0006	1.00	0.0010	−0.50
China	Xishuangbanna	20	71	0.0144	0.99	0.0034	0.83
Indonesia	East Java	11	10	0.0022	0.93	0.0012	0.56
Japan	Aichi	6	3	0.0015	1.00	0.0023	−0.06
	Iriomote	1	NA	NA	NA	0.0022	0.00
	Okinawa	12	900	0.2742	0.76	0.0923	0.65
Taiwan	Dali	6	1059	0.2845	0.77	0.0052	0.98
Thailand	Doi Pui	4	0	0.0000	1.00	0.0000	NA
Honduras	Atlántida	3	0	0.0004	1.00	0.0007	−0.50
United States	Arkansas	5	23	0.0082	0.99	0.0019	0.75
	Florida	24	98	0.0211	0.98	0.0040	0.81
	Georgia	22	131	0.0361	0.97	0.0141	0.60
	Hawaii	7	1064	0.3638	0.68	0.0007	1.00
	Maryland	8	300	0.0342	0.97	0.0315	0.03
	North Carolina	12	78	0.0142	0.99	0.0013	0.93
	South Carolina	4	69	0.0252	0.98	0.0158	0.37
	Virginia	20	28	0.0050	1.00	0.0014	0.82
Papua New Guinea	Mandang	7	1	0.0005	1.00	0.0009	0.00
Overall		198	1365	0.0579	0.59	0.0091	0.8429

**Table 3:** Genotype and heterozygous genotype frequencies at each location and averaged per individual

Location	*n*	Polymorphic loci	Heterozygous loci	Genotypes per location (%)	Heterozygous genotypes per location (%)	Genotypes per individual	Heterozygous genotypes per individual
Cameroon	7	3	2	9346 (98)	3 (0.03)	1335 (±43 *SD*)	0.4 (±0.5 *SD*)
Ghana	11	3	2	14742 (98)	4 (0.02)	1339 (±46 *SD*)	0.4 (±0.5 *SD*)
Madagascar	8	1	2	10801 (99)	10 (0.09)	1350 (± 12 *SD*)	1.3 (±0.5 *SD*)
China	20	71	43	26341 (97)	104 (0.40)	1317 (±122 *SD*)	4.2 (±3.4 *SD*)
Indonesia	11	10	3	14005 (93)	15 (0.11)	1273 (±165 *SD*)	1.4 (±0.8 *SD*)
Aichi, Japan	6	3	2	6611 (81)	13 (0.19)	1101 (±94 *SD*)	2.2 (±0.4 *SD*)
Iriomote, Japan	1	NA	3	1351 (99)	3 (0.22)	NA	NA
Okinawa, Japan	12	900	965	15034 (92)	1363 (9.07)	1253 (±91 *SD*)	112.6 (±253.7 *SD*)
Taiwan	6	1059	31	7657 (93)	36 (0.47)	1276 (±91 *SD*)	6.0 (±6.6 *SD*)
Thailand	4	0	0	4526 (83)	0	1132 (±171 *SD*)	0
Honduras	3	0	1	4015 (98)	2 (0.05)	1338 (±33 *SD*)	0.7 (±0.6 *SD*)
Arkansas, United States	5	23	6	6709 (98)	11 (0.16)	1341 (±48 *SD*)	2.2 (±1.3 *SD*)
Florida, United States	24	98	44	32760 (95)	146 (0.47)	1291 (±98 *SD*)	5.1 (±9.4 *SD*)
Georgia, United States	22	131	116	27033 (99)	407 (1.51)	1353 (±43 *SD*)	18.9 (±12 *SD*)
Hawaii, United States	7	1064	3	9555 (98)	6 (0.06)	1337 (±41 *SD*)	0.8 (±1.1 *SD*)
Maryland, United States	8	300	292	9487 (87)	303 (3.20)	1186 (±182 *SD*)	37.9 (±100 *SD*)
North Carolina, United States	12	78	8	16086 (98)	31 (0.19)	1341 (±42 *SD*)	1.6 (±1.8 *SD*)
South Carolina, United States	4	69	42	5417 (99)	83 (1.53)	1354 (±10 *SD*)	20.8 (±15.1 *SD*)
Virginia, United States	20	28	12	27093 (99)	56 (0.21)	1356 (±22 *SD*)	1.8 (±1.8 *SD*)
Papua New Guinea	7	1	2	8923 (93)	5 (0.06)	1275 (±56 *SD*)	0.7 (±0.5 *SD*)
Overall	198	1365	1056	258419 (96)	2719 (1.05)	1307 (100 *SD*)	13.1 (68 *SD*)

### 3.2 Population structure and history

**Figure 3:**
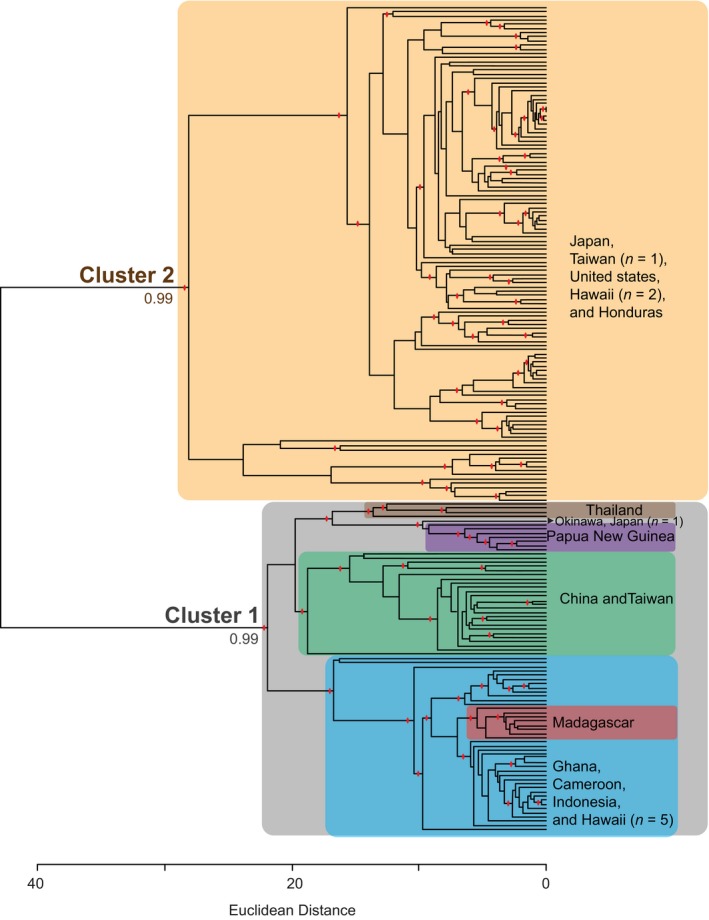
Ultrametric dendrogram of hierarchical clusters for all individuals. Statistically significant clusters (*p* < .05) are indicated by read hatch marks at cluster nodes. Clusters containing all individuals from one location are highlighted with color and labeled

In its native East Asia, *X. crassiusculus* is comprised of at least two separate populations, sampled here: one in Southeast Asia including the mainland China, Thailand, and Taiwan, and one on most Japanese islands (Figure 2). Okinawa and Taiwan contain individuals from each population. The rest of the world was likely colonized from these two populations or a similarly closely related unsampled population: North America and Central America from the Japanese populations, Africa and Oceania from the mainland. Hawaii has been colonized by both populations. This is indicated foremost by hierarchal clustering where two highly divergent and very well supported (*p* < .001) primary clusters are observed (Figure 3) and secondarily by Bayesian clustering (*K* = 2, ∆*K* = 118021.52, Table 4). These two most inclusive clusters, hereafter referred to as cluster 1 and cluster 2, encompassed all 198 individuals. Both clusters included individuals from the beetles' native range. Cluster 1 contained exclusively native‐range beetles from China and Southeast Asia, and cluster 2 primarily contained native‐range beetles from Japan suggesting cluster origin, respectively. Cluster 1 also included all nonnative‐range individuals from Taiwan, Southeast Asia, Africa, and five individuals from Hawaii, while cluster 2 included all introduced individuals from the continental United States, two individuals from Hawaii, and all individuals from Honduras. The genetic divergence between these two clusters is the largest source of genetic variation (61.2%, *p* < .01; Table 5) among the beetles surveyed here. The clustering appears robust, as individuals were correctly reassigned to their respective clusters 100% of the time in the Discriminate Analysis of Principal Components.

**Table 4:** Bayesian clustering results for clusters *K* = 1–16 with five replicates

*K*	Reps	Mean LnP(*K*)	Stdev LnP(*K*)	Ln′(*K*)	|Ln′′(*K*)|	∆*K*
1	5	−304553.5	0.8	NA	NA	NA
2	5	−71366.44	1.8889	233187.06	222932.62	118021.5183
3	5	−61112.00	4436.6548	10254.44	69.2	0.015597
4	5	−50926.76	4522.6007	10185.24	4033.3	0.89181
5	5	−44774.82	8771.3327	6151.94	6925.4	0.789549
6	5	−45548.28	9325.2556	−773.46	8152.38	0.874226
7	5	−38169.36	6391.3703	7378.92	7406.34	1.158803
8	5	−38196.78	6362.8556	−27.42	3994.26	0.627746
9	5	−34229.94	1907.2667	3966.84	3303.74	1.732186
10	5	−33566.84	7.6045	663.1	608.84	80.063411
11	5	−33512.58	423.8905	54.26	227.6	0.536931
12	5	−33685.92	497.6567	−173.34	498.82	1.002338
13	5	−33360.44	85.714	325.48	377.58	4.405114
14	5	−33412.54	116.7327	−52.1	318.8	2.731027
15	5	−33783.44	506.9328	−370.9	625.72	1.234325
16	5	−33528.62	353.905	254.82	NA	NA

**Table 5:** Hierarchical analysis of molecular variance (AMOVA) to test for the effects of clusters (determined here) or location on the genetic diversity of *Xyleborus crassiusculus*. Φ_CT_ is the differentiation between clusters, Φ_SC_ is differentiation among populations within clusters, and Φ_ST_ is differentiation among populations

Source	Variance	Variance (%)	*p* value	Φ_CT_	Φ_SC_	Φ_ST_
Between clusters	13.12	61.20	<.01	0.61		
Among locations within clusters	4.40	20.55	<.01		0.53	
Among locations	3.91	18.25	<.01			0.82

**Figure 4:**
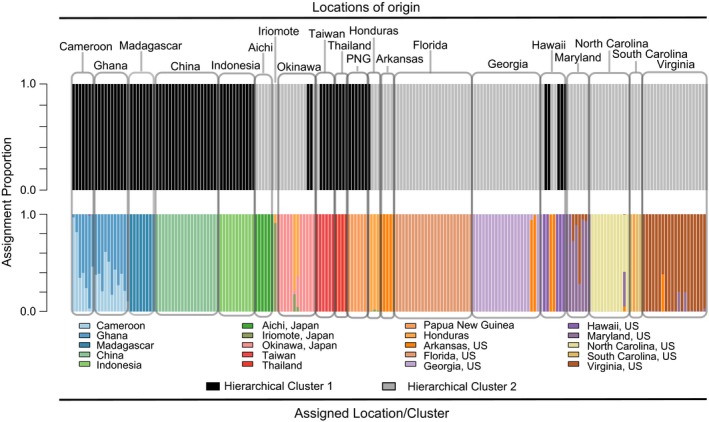
Probability of reassignment of each individual to its location of origin (bottom) and to the two primary hierarchical clusters determined here (top)

The two global East Asian populations identified here were not the only source of genetic differentiation in *X. crassiusculus*. Across all populations, there were seventy additional statistically significant hierarchical clusters (Figure 3) and genetically distinct subpopulations roughly associated with geographic location. In cluster 2, which contained native beetles from Japan, the majority of subclusters contained either a few individuals from one location or several individuals from different locations. For example, beetles from Maryland and Virginia were not always genetically discernible from each other when reassigned back to locations of origin in the DAPC, while beetles from Florida could be accurately (>99%) reassigned to their location of origin (Figure 4). In contrast, the global population of Chinese origin is composed of much more homogeneous subclusters. Papua New Guinea, Thailand, and Madagascar all formed location‐specific subclusters. There were also two larger subclusters: one contained all individuals from China and Taiwan, and the other included individuals from Africa, Indonesia, and Hawaii. In Africa, Ghana and Cameroon likely represent either a single or admixed population (Figure 4) because little differentiation was detected between individuals at these locations. High levels of genetic differentiation were also detected between some subclusters (F_ST_ > 0.8) and between locations within subclusters (Table 6).

**Table 6:** Pairwise F_ST_ values between locations. Locality row labels have been abbreviated and are ordered as columns

	Cameroon	Ghana	Madagascar	Thailand	Indonesia	PNG	China	Taiwan	Okinawa	Hawaii	Aichi	Iriomote	Honduras	Florida	Georgia	Arkansas	North Carolina	South Carolina	Virginia	Maryland
Cam	0.00																			
Gha	0.02	0.00																		
Mad	0.92	0.93	0.00																	
Tha	0.83	0.84	0.82	0.00																
Ind	0.89	0.91	0.87	0.82	0.00															
PNG	0.80	0.83	0.81	0.17	0.82	0.00														
Chi	0.86	0.89	0.87	0.55	0.89	0.71	0.00													
Tai	0.39	0.43	0.39	0.18	0.44	0.23	0.23	0.00												
Oki	0.53	0.59	0.55	0.36	0.59	0.42	0.62	0.28	0.00											
Haw	0.17	0.20	0.19	0.22	0.25	0.27	0.43	0.14	0.23	0.00										
Aic	0.70	0.75	0.72	0.47	0.75	0.54	0.73	0.31	0.06	0.26	0.00									
Iri	0.99	0.99	0.99	0.92	0.98	0.82	0.84	0.39	0.09	0.25	0.00	0.00								
Hon	1.00	1.00	1.00	0.96	0.99	0.93	0.94	0.59	0.20	0.45	0.50	0.00	0.00							
Fla	0.95	0.96	0.95	0.88	0.96	0.91	0.96	0.72	0.43	0.64	0.68	0.10	0.26	0.00						
Geo	0.91	0.94	0.92	0.82	0.93	0.87	0.94	0.67	0.36	0.59	0.60	0.06	0.18	0.39	0.00					
Ark	0.99	0.99	0.99	0.96	0.99	0.94	0.95	0.66	0.27	0.52	0.61	0.69	0.86	0.50	0.13	0.00				
NorC	0.98	0.98	0.98	0.94	0.98	0.94	0.96	0.72	0.37	0.61	0.69	0.36	0.63	0.56	0.16	0.25	0.00			
SouC	0.98	0.98	0.98	0.94	0.98	0.92	0.94	0.62	0.23	0.49	0.55	0.21	0.40	0.31	0.12	0.58	0.40	0.00		
Vir	0.99	0.99	0.99	0.96	0.99	0.96	0.98	0.75	0.44	0.65	0.75	0.54	0.78	0.72	0.33	0.38	0.53	0.62	0.00	
Mar	0.96	0.96	0.96	0.91	0.96	0.91	0.94	0.67	0.32	0.56	0.62	0.30	0.53	0.49	0.17	0.16	0.26	0.40	0.20	0.00

At most locations, heterozygosity was low (<0.1; Table 2) as heterozygous genotypes were rare. The proportion of shared alleles was high (>0.9) except within the Taiwan, Okinawa, and Hawaii populations, which all had the lowest proportion of shared alleles (<0.8), in addition to large numbers of SNPs, and the highest gene diversities (>0.2). Some locations such as Maryland also had large numbers of SNPs, but gene diversity was low and the proportion of shared alleles high (>0.9). Inbreeding, as indicated by the inbreeding coefficient *F*_IS_, was very variable, ranging from −0.5 to 1.0 (Table 2). While inbreeding is clearly evident given the scarcity of heterozygous genotypes and often high inbreeding coefficients, outbreeding is also common at some locations as evidenced by the proportion of shared alleles, the higher than expected variation in inbreeding coefficients and even low *F*_IS_. In several populations, outbreeding appears to occur regularly (Madagascar, Aichi, and Honduras). These populations are characterized by negative *F*_IS_ values, low numbers of polymorphic sites (<3), and high proportion of heterozygous sites relative to SNPs (Table 2). However, given the few number of polymorphic loci at these locations, it is possible that the negative inbreeding indices reflect the high proportion of heterozygous polymorphic loci. For example, the single polymorphic loci in the population sample from Madagascar are heterozygous (Table 3).

## 4 DISCUSSION

Populations of *X. crassiusculus* in invaded regions display significant population heterogeneity, particularly in the well‐sampled North America. It is unclear whether this is a result of a few independent introductions. However, this is unlikely, as in that case, we would expect to observe several distinct, clonal, or near‐clonal populations in North America. The more likely explanations include (1) numerous, repeated introductions that blur between‐group differences, or (2) introduction of a large founding population that retained its genetic variation, or (3) any of the above scenarios with a more than expected degree of outcrossing. All three scenarios are also mutually compatible.

Human‐mediated expansion is the most likely route of introduction for *X. crassiusculus* especially at most U.S. locations given the documented continuous invasion of nonnative Xyleborini species through coastal areas (Rassati et al., 2016) and due to the fact that the species was simply absent in most of the sampled areas outside of Asia just a few decades ago. However, it is also possible that some expansion within the Old World has occurred without human‐aid (Gohli et al., 2016). Historic dispersal is possible in *X. crassiusculus* where genetic divergence is high between nearby locations, for example, in Madagascar which is highly differentiated from all other African and Asian locations (Table 6).

It has been traditionally assumed that Xyleborini ambrosia beetles are functionally clonal, because haploid, flightless, and blind males are confined to their native galleries and unlikely to facilitate any gene flow between families. This has been revised by recent observations suggesting that males actually routinely crawl out of their native galleries in search for additional mating opportunities on the same tree (Peer & Taborsky, 2004). Indeed, at all of our locations, there are individuals with estimated inbreeding indices lower than expected for a completely inbred species, and this observation is driven by a few heterozygous genotypes. In fact, in a few locations, heterozygosity is uncharacteristically high for an inbreeding organism. These findings are contrary the traditional assumption of complete inbreeding in this species and call for reassessment of these assumptions in other putatively inbred invasive species.

The robust differentiation between the Japanese and mainland Asia beetle lineages might indicate cryptic speciation. High intraspecific genetic differences in *X. crassiusculus* have been observed among a few individuals sequenced at the COI locus (Dole et al., 2010). However, testing for cryptic speciation is beyond the scope of this study, and it is not even clear whether organisms with predominant inbreeding conform to any of the standard species concepts (see Fontaneto et al. (2007) for discussion). The significant substructure within the mainland lineage (cluster 1) might indicate that these are older populations that began to diverge earlier, or that the Japanese populations have not been sufficiently sampled.

Unsampled native‐range populations could also be source populations. Given the high level of differentiation between lineages observed here and by others (Dole et al., 2010), we hypothesize that any unsampled populations would cluster within these two lineages. This can be tested for in the future using approximate Bayesian computation, which enables tests of multiple introduction scenarios, such as historic and recent, local expansion in introduced ranges, and admixture.

*Xylosandrus crassiusculus* continues to spread around the world. Even during the preparation of this manuscript, a new expanding population has been reported from South America (Flechtmann & Atkinson, 2016). It is not the only insect with a rapidly expanding range. There are dozens of bark and ambrosia species, and thousands of species from other groups that are being introduced by human transport to nonnative locations. Communities of wood‐boring insects around the world are beginning to resemble one another, and differences between regional assemblages fade (Brockerhoff, Bain, Kimberley, & Knížek, 2011; Haack, Rabaglia, & Peña, 2013). Global biotic homogenization is causing not only economic and ecological damage via the introductions of invasive species, but also the loss of biogeographic history. Increasingly, variation inside organisms' genomes is the only evidence of the past geographical diversity of animals and plants on the planet. Here, we show that affordable and effective analytical approaches are now available to disentangle the complex global structure of tramp species, even those with unusual reproduction strategies and even in the absence of any previous population or genome data.

## CONFLICT OF INTEREST

None declared.

## AUTHOR CONTRIBUTIONs

Caroline Storer contributed sample acquisition, specimen preparation, sequencing, analyses, writing, and interpretation. Adam Payton contributed to project design, analyses, writing, and interpretation. Stuart McDaniel contributed to project design, data acquisition, and interpretation. Bjarte Jordal contributed to sample acquisition and interpretation. Jiri Hulcr contributed to project design, sample and data acquisition, interpretation, and writing.

## DATA ACCESSIBILITY

Unfiltered barcode‐split ddRADseq reads with quality scores for each individual have been deposited in the GenBank Short Read Archive (SRA), accession numbers SRR4181068‐265 under BioProject PRJNA342041. Analyses were performed using publicly available scripts and packages described in the methods.
